# The Effectiveness of Antipsychotic Drug Therapy for Treating Psychosis in People With Epilepsy – a Systematic Review

**DOI:** 10.1192/bjo.2024.108

**Published:** 2024-08-01

**Authors:** Aryan Arora, Priya Prakash, Laura Rizzo, Jonathan Rogers

**Affiliations:** UCL, London, United Kingdom

## Abstract

**Aims:**

Individuals with epilepsy are at risk of developing pre-ictal, ictal, postictal or interictal psychoses. Antipsychotic drugs (APDs) are the main class of drugs used to treat psychosis and schizophrenia. The efficacy and safety of APDs as a treatment for epileptic psychosis is not well understood. Hence, we aimed to conduct a systematic review assessing the effectiveness and adverse effects of antipsychotic drugs to treat psychosis in people with epilepsy.

**Methods:**

We adhered to the Preferred Reporting Items for Systematic Reviews and Meta-Analyses (PRISMA) guidelines. We searched MEDLINE, Embase, PsycInfo and AMED from database inception to 20/06/2023. We contacted experts in the field and performed citation searches to identify additional records. Title, abstract, full-text review, and data analysis were conducted in duplicate, with conflicts resolved by discussion among authors. Given the heterogeneity of study designs, meta-analysis was not deemed appropriate; instead, the results were tabulated in a narrative synthesis. The Joanna Briggs Institute Risk of Bias tool was used to assess study quality.

**Results:**

We identified 13 studies, with a total of 1,180 participants. In the 9 case series included, the psychotic symptoms of all but 3 out of 28 patients treated with APDs partially improved or fully resolved. 3 of the cohort studies reported an association between antipsychotic use and longer duration of psychotic episodes, 2 found similar results in both APD and non-APD groups, and 2 did not report control psychosis outcomes. When reported, seizure frequency was observed to remain unchanged or decrease following APD treatment.

**Conclusion:**

Available evidence does not suggest that antipsychotics increase seizure risk in individuals with epilepsy. However, further data from randomised controlled trials and well-controlled cohort studies are urgently needed to draw more definitive conclusions.

